# Dysfunction and Metabolic Reprogramming of Gut Regulatory T Cells in HIV-Infected Immunological Non-Responders

**DOI:** 10.3390/cells14151164

**Published:** 2025-07-29

**Authors:** Minrui Yu, Mengmeng Qu, Zerui Wang, Cheng Zhen, Baopeng Yang, Yi Zhang, Huihuang Huang, Chao Zhang, Jinwen Song, Xing Fan, Ruonan Xu, Yan-Mei Jiao, Fu-Sheng Wang

**Affiliations:** 1 Medical School of Chinese PLA, Beijing 100853, China; ymr121331@163.com; 2 Senior Department of Infectious Diseases, The Fifth Medical Center of Chinese PLA General Hospital, National Clinical Research Center for Infectious Diseases, Beijing 100039, China; qumm302@163.com (M.Q.); zhencheng302@126.com (C.Z.); g8_1980530@outlook.com (B.Y.); yiyixiaoyiya@163.com (Y.Z.); hhh-878766@163.com (H.H.); zhangch302@163.com (C.Z.); songjinwenchina@yeah.net (J.S.); fanxing302@aliyun.com (X.F.); xuruonan2004@aliyun.com (R.X.); 3 Senior Department of Gastroenterology, The First Medical Center of Chinese PLA General Hospital, Beijing 100853, China; wzr301gh@163.com

**Keywords:** HIV, immunological non-responders, immunological responders, Tregs, gut, SCFA

## Abstract

Disruption of the gut microenvironment is a hallmark of HIV infection, where regulatory T cells (Tregs) play a critical role in maintaining gut homeostasis. However, the mechanisms by which gut Tregs contribute to immune reconstitution failure in HIV-infected individuals remain poorly understood. In this study, we employed single-cell RNA sequencing (scRNA-seq) to analyze gut Treg populations across three cohorts: eight immunological responders (IRs), three immunological non-responders (INRs), and four HIV-negative controls (NCs). Our findings revealed that INRs exhibit an increased proportion of gut Tregs but with significant functional impairments, including reduced suppressive capacity and heightened apoptotic activity. Notably, these Tregs underwent metabolic reprogramming in INRs, marked by an upregulation of glycolysis-related genes and a downregulation of the oxidative phosphorylation (OXPHOS) pathway. Additionally, both the abundance of short-chain fatty acid (SCFA)-producing bacteria and SCFA concentrations were reduced in INRs. In vitro SCFA supplementation restored Treg function by enhancing suppressive capacity, reducing early apoptosis, and rebalancing cellular energy metabolism from glycolysis to OXPHOS. These findings provide a comprehensive characterization of gut Treg dysfunction in INRs and underscore the therapeutic potential of targeting gut Tregs through microbiota and metabolite supplementation to improve immune reconstitution in HIV-infected individuals.

## 1. Introduction

Currently, antiretroviral therapy (ART) is the standard treatment for people living with HIV (PLWH), proving effective in suppressing HIV replication, improving immune function, and significantly reducing the mortality rate [1]. However, despite achieving undetectable viral load with long-term ART, a significant proportion of PLWH (10–40%) fail to achieve adequate immune reconstitution [2]. These individuals are referred to as immunological non-responders (INRs), in contrast to immunological responders (IRs). INRs face a higher risk of developing both AIDS-related and non-AIDS-related illnesses, as well as increased mortality [3,4]. Although previous studies have explored the mechanisms underlying immune reconstitution failure, the precise factors remain elusive, hindering the development of effective clinical interventions.

The gut, rich in lymphoid tissue and lymphocytes, is a major site for HIV transmission, pathogenesis, and persistence, making it one of the earliest and most vulnerable targets for the virus [5,6]. While ART effectively promotes immune reconstitution in peripheral blood, immune recovery within the gastrointestinal (GI) tract is markedly delayed, highlighting a critical disparity in immune reconstitution. Even with prolonged and optimized ART regimens, mucosal immune recovery remains incomplete, underscoring a limitation of current therapeutic strategies [7,8]. Consequently, GI tract damage occurs early in the infection, is often irreversible, and persists throughout the course of HIV infection [9]. Microbiota translocation leads to systemic inflammation and increases the risk of non-AIDS-related morbidity and mortality among HIV-infected individuals [10,11,12,13]. Our previous study found that INRs exhibit more severe gut damage compared to IRs [14]. Moreover, research indicates that toxic solutes originating in the gut bacterial flora could potentially hinder CD4^+^ T cell recovery on ART and contribute to the CD4^+^ T cell lymphopenia observed in INRs [15]. Prior studies indicate probiotic supplementation increases T lymphocytes and confers immunological/virological benefits in ART-treated PLWH [16]. Thus, many aspects of gut microenvironment disruption and its role in immune reconstitution failure remain to be further understood.

Regulatory T cells (Tregs) are a unique cell lineage of adaptive immune system, critical for maintaining immune system balance and protecting against autoimmune and inflammatory disorders [17]. Tregs exhibit a dual role in HIV pathogenesis: while their immunosuppressive action impairs antiviral immune responses, facilitating viral persistence, they also mitigate HIV-associated immune activation, a hallmark of disease progression [18]. There are contradictory results regarding the number and the role of Tregs in the progression of HIV/AIDS among both untreated PLWH and those receiving ART [19]. The discrepancy in various research results may stem from several factors, including the heterogeneity of the HIV-infected population being studied, diverse markers used to define Tregs, and the inherent heterogeneity of Tregs across tissues [20,21]. Most current research focuses on peripheral blood analysis, yet our previous study suggests a significant increase in the percentage of gut Tregs in INRs [14]. Unlike peripheral blood Tregs, gut Tregs exhibit unique functional diversity and play a pivotal role in maintaining intestinal homeostasis. This specialized microenvironment, continuously exposed to diverse microbes and food antigens, establishes a dynamic relationship: Tregs regulate microbial composition and diversity, while the gut microbiota shapes Treg development, differentiation, and function [22,23,24]. Consequently, Tregs are crucial for regulating immune responses, preventing excessive reactions to common antigens, mediating immune tolerance [25], and maintaining gut mucosal barrier integrity [26,27,28]. However, data on the relationship between gut Treg changes and immune reconstitution in HIV-infected individuals, particularly INR, are limited. In recent years, the emergence of single-cell RNA sequencing (scRNA-seq) technology has significantly advanced the study of complex immune system functions and regulatory mechanisms. Unlike conventional bulk RNA sequencing, scRNA-seq provides high-resolution transcriptomic profiling at the single-cell level. This capability allows researchers to dissect cellular heterogeneity and, crucially, to reveal the distinct characteristics of Tregs in gut tissue [29,30,31,32].

It is well-established that HIV-infected individuals exhibit alterations in gut microbiota diversity and composition, with gut microbiota dysbiosis and microbial translocation closely linked to disease progression [33,34,35]. Short-chain fatty acids (SCFAs), major metabolites derived from gut microbiota, are linked to diet, microbiota, and host immune system and gut homeostasis. SCFAs have been reported to regulate gut Treg size and function, promoting gut homeostasis [36,37]. SCFA supplementation has emerged as a promising therapeutic strategy to reduce immune activation and inflammation in multiple human diseases [38,39]. However, whether SCFA levels differ between INRs and IRs and their effect on Tregs in HIV-infected individuals remains unknown.

To investigate the characteristics of gut Tregs in HIV-infected individuals and explore the underlying mechanisms, we employed scRNA-seq to analyze the immune profiles of gut Tregs from IRs, INRs, and HIV-negative controls (NCs). Additionally, we examined the correlations between the abundance of SCFA-producing microbiota, SCFA concentrations, and the proportion of gut Tregs. An in vitro culture system was utilized to clarify the role of SCFAs in regulating Treg cell metabolism during HIV immune reconstitution. Our findings highlight the characteristics of gut Tregs that contribute to immune recovery in INRs, which may provide guidance for the development of immunotherapies.

## 2. Materials and Methods

### 2.1. Study Subjects and Sample Processing

Ileocyte mucosal tissue samples were collected from 11 male participants with chronic HIV infection. All participants had undergone ART for >2.5 years and achieved persistent undetectable plasma HIV RNA after 6 months of ART. Based on peripheral CD4 T cell counts, 3 were classified as immunological non-responders (INRs; CD4 T cell counts < 200 cells/µL) and 8 as immunological responders (IRs; CD4 T cell counts > 500 cells/µL) [40,41]. HIV-negative gut samples were collected from 4 individuals who underwent gut examinations as NCs. Exclusion criteria included (1) evident gut diseases; (2) concurrent active opportunistic infections; (3) concomitant tumors; (4) organ dysfunction or decompensation; (5) refusal to cooperate. The peripheral blood samples were obtained from 44 participants (14 NCs, 16 IRs and 14 INRs) for in vitro experiments. This research was approved by the Ethics Committee of the Fifth Medical Center of the Chinese PLA General Hospital. The detailed characteristics of the enrolled subjects are listed in Table 1 (ileocyte mucosal tissue samples) and Supplemental Table S1 (Peripheral Blood Mononuclear Cells, PBMCs).

Ileocecal tissue was collected during colonoscopy and immediately preserved. The tissue was then cut into 1–2 mm fragments and digested with 0.1% collagenase IV for 30 min under shaking. To terminate the digestion, culture medium was added. The resulting mixture was passed through a 70 μm cell strainer to obtain a cell filtrate. Red blood cells were lysed and the pellet was washed. The resulting suspension was filtered through a 40 μm cell strainer. Cells were counted, and viability was confirmed to be >85% before proceeding to subsequent analysis [42,43]. PBMCs were isolated from whole blood using Ficoll-Paque™ PLUS (Cytiva, Grens, Sweden) according to the manufacturer’s instructions. Freshly isolated PBMCs were stained with fluorochrome-conjugated monoclonal antibodies against human CD3 (OKT3; BioLegend, California, USA), CD4 (RPA-T4; BioLegend, California, USA), CD45 (HI30; BioLegend, California, USA), CD127 (A019D5; BioLegend, California, USA) and CD25 (2A3; BD Biosciences, New Jersey, USA), along with Fixable Viability Stain 780 (BD Biosciences, New Jersey, USA) for live/dead discrimination. Tregs were identified as CD3^+^ CD45^+^ CD4^+^ CD25^high^ CD127^low/−^ populations and subsequently sorted using fluorescence-activated cell sorting (FACS). The PBMCs were resuspended in FACS buffer (1 × PBS + 2% bovine serum albumin) and sorted by FACS (Sony, MA900, Tokyo, Japan). Representative images of flow cytometry sorting strategy are shown in Supplementary Figure S3. The purity of each isolated Tregs was generally above 95%. The sorted cells were cultured for further in vitro assay.

### 2.2. ScRNA-Seq Library Construction, Read Mapping and Quality Control

Library preparation and sequencing were conducted on the NovaSeq 6000 platform (Illumina, Inc., San Diego, CA, USA) with the support of Shanghai Biotechnology Corporation (Shanghai, China). The GRCh38-2020 was integrated into the CellRanger (version 7.2.0) pipeline as a transcriptome reference for alignment. The ‘Cell Ranger count’ function generated gene counts for a single library, managing alignment, filtering, and UMI counting. Data processing was further conducted using the Seurat V4 package in R (version: 4.3.2).

Genes expressed in more than 0.1% of cells and cells with more than 200 detected genes were selected for deeper analysis. Low-quality cells were excluded based on metrics including fewer than 1000 UMIs, less than 500 detected genes, or over 10% of UMIs derived from mitochondrial genes. To mitigate batch effects stemming from sequencing batches and other variables, the Harmony (version 1.2.0) function was utilized for normalization.

### 2.3. ScRNA-Seq Data Processing

After removing low-quality cells, the gene expression matrix was normalized using the NormalizeData function. The FindVariableFeatures function was then used to identify 2000 highly variable genes across cells. The data were linearly transformed and scaled using the ScaleData function with default parameters, followed by dimensionality reduction via the RunPCA function. Following these steps, the Elbowplot, DimHeatmap, and JackStrawPlot functions, as recommended by Seurat developers, were used to accurately determine the true dimensionality of each dataset. Cell clustering was subsequently performed using the FindNeighbors and FindClusters functions, followed by nonlinear dimensionality reduction via the RunUMAP function with default parameters. For cell-type-specific analyses, a resolution of 0.1 was applied to CD4^+^ T cells and 0.4 to Tregs.

### 2.4. Cell Annotation and Subpopulation Re-Analysis

The primary annotation of major cell types was based on classical markers, with significant reference to CellMarker 2.0 [44]. Building upon our previous annotation, the CD4^+^ T cell population was further subsetted for analysis using dimensionality reduction and clustering technique [42]. Within the CD4^+^ T cell compartment, Tregs were identified based on classical markers, including *ICOS*, *FOXP3*, and *CTLA4*, allowing clear distinction from non-Tregs. These Tregs underwent further dimensionality reduction and clustering analyses to better characterize their subpopulations.

### 2.5. Cell Distribution Discrepancy

For each participant, the count of each cell type was normalized to the total cell count, and a stacked plot was used to visualize the proportional distribution of cell types. Additionally, Ro/e analysis was conducted to identify discrepancies in cell distribution across different groups. The Ro/e analysis compares the observed and expected cell counts, thereby quantifying each subgroup’s tendency to reside in the tissue. Generally, a Ro/e ratio greater than 1.5 indicates enrichment within the tissue, whereas a ratio below 0.5 suggests depletion.

### 2.6. Differentially Expressed Gene (DEG) Analysis

DEGs were identified using the FindMarkers function in Seurat, which employs the default Wilcoxon test (test.use = “wilcox”). Genes were considered significant if |log2 fold change| > 0.25 and *p*-value < 0.05. A volcano plot was generated to visualize these DEGs across pairwise comparison groups. Additionally, a heatmap was used to display significant genes from each group, illustrating their expression patterns.

### 2.7. Functional Scoring

Cellular functional states, including proliferation, apoptosis, TGF-β signaling, and IL-10 production, were assessed using the AddModuleScore function in the Seurat package.

This function evaluates the activity or expression levels of predefined gene sets at the single-cell level in scRNA-seq data. The specific gene sets used in this analysis are provided in Supplementary Table S2.

### 2.8. Trajectory Analysis

The trajectory of Treg subpopulations was analyzed using the R package Monocle (version 2.26.0). Initially, the UMI matrix was imported from the Seurat object, and a new dataset was created using the “newCellDataSet” function. Highly variable genes among Treg subtypes were identified using the “dispersionTable” function. Dimensionality reduction was performed using the “reduceDimension” function with the “DDRTree” method. Finally, cells were ordered based on predicted pseudotime using the “orderCells” function.

### 2.9. Faecal Microbiota Analysis

Microbial genomic DNA was extracted from faecal samples using the E.Z.N.A.^®^ Soil DNA Kit (Omega Bio-tek, Norcross, GA, USA) according to the manufacturer’s instructions. Paired-end libraries were then constructed using the NEXTFLEX Rapid DNA-Seq Kit (Revvity, Massachusetts, USA). Metagenomic sequencing was conducted on the Illumina NovaSeq platform, provided by Wuhan Bio-Broad Biotech Co., Ltd., Wuhan, China. Following data quality control, assembly, and gene set construction, species abundance in the gut microbiota was analyzed using Diamond software (version 0.8.35).

### 2.10. HIV DNA and CA HIV RNA Quantification

Total DNA was extracted from PBMCs and gut mucosal tissues using the QIAamp DNA Mini Kit (Qiagen, Hesse, Germany) and the Qiagen DNeasy Blood & Tissue Kit (Qiagen, Hesse, Germany), respectively. Total RNA was extracted from both gut mucosal tissues and PBMCs using the Hi-Pure Total RNA Plus Mini Kit (MGBio, Shanghai, Beijing). After extraction, real-time PCR was performed to amplify the target sequences. Finally, parameters were adjusted based on off-machine data to accurately quantify HIV DNA and CA HIV RNA levels per million PBMCs or total cells, as previously reported [35].

### 2.11. Detection of Plasma Short-Chain Fatty Acids

To explore the variations in the types and concentrations of SCFAs in plasma samples, we utilized chromatography–mass spectrometry for quantification. Chromatographic separation was accomplished using a BEH C18 column, employing a linear gradient elution mode. The detection and analysis were performed on an Agilent 1290 ultra-high-performance liquid chromatography (UHPLC) system (Agilent, California, USA), integrated with an Agilent 6470 triple quadrupole mass spectrometer. The raw data were processed with MassHunter software (version B.08.00, Agilent, California, USA), which provided chromatographic peak area data for each compound and the internal standard. The concentrations of the target compounds in the test samples were subsequently calculated using quantitative calibration curves and reported.

### 2.12. In Virto Stimulation

The enriched Tregs were cultured in 96-well U-bottom plates under conditions of human recombinant IL-2 (100 IU/mL) and anti-CD3/CD28 antibodies (5 µg/mL) at a concentration of 1 × 10^6^ cells/mL, following detailed protocols outlined in prior studies [45,46,47]. To examine the functional effects of SCFAs on Tregs, the enriched Tregs were either unstimulated or stimulated with a combination of SCFAs, namely sodium acetate, sodium propionate, and sodium butyrate, for 3 days. We employed both low and high SCFA concentrations as specified in previous research [48,49]. Following the incubation period, both the cultured Tregs and the supernatants were harvested for further analysis.

### 2.13. Detection of Cell Apoptosis and Cytokines

To analyze cytokines in the supernatants of Treg cultures, we employed the Human Th1/Th2 6-plex Panel 1 and TGF-β1 1-plex kits (Kuangbo Biosciences, Beijing, China). All assays were performed in accordance with the manufacturer’s protocols, which included the utilization of specific standards and quality control samples. Cytokines were quantified using flow cytometry. To assess apoptosis in Tregs, the APC Annexin V Apoptosis Detection Kit with 7-AAD (BD Biosciences, New Jersey, USA) was utilized. The cells were resuspended in binding buffer, incubated with the APC Annexin V and 7-AAD Viability Staining Solution on ice for 15 min, and subsequently analyzed by flow cytometry within 1 h.

### 2.14. Detection of Cellular Energy Metabolism

To assess the changes in energy metabolites produced by Tregs upon stimulation by SCFAs, an Agilent 1290 Infinity LC ultra-high-performance liquid chromatography (UHPLC) system (Agilent, California, USA) was utilized for metabolite separation. Mass spectrometry analysis was conducted using a 5500 QTRAP mass spectrometer (SCIEX, California, USA) operating in negative ion mode. Standard compounds were employed as references to detect key metabolites involved in energy metabolic pathways, including glycolysis and OXPHOS.

### 2.15. Statistical Analysis

Statistical analyses were conducted using the R software (version: 4.3.2). For two-group comparisons, the Mann–Whitney U test was performed for unpaired samples, while the Wilcoxon signed-rank test was applied for paired samples. For analyses involving three or more groups, an initial global analysis was conducted utilizing the Kruskal–Wallis test for unpaired samples. If significant global differences were observed (*p* < 0.05), the Mann–Whitney U test was subsequently performed. Correlation analyses were carried out using Spearman’s rank correlation coefficient. A *p* value of <0.05 was considered indicative of statistical significance for all tests.

## 3. Results

### 3.1. Marginal Increase but Dysfunction of Gut Tregs in INRs

**Figure 1:**
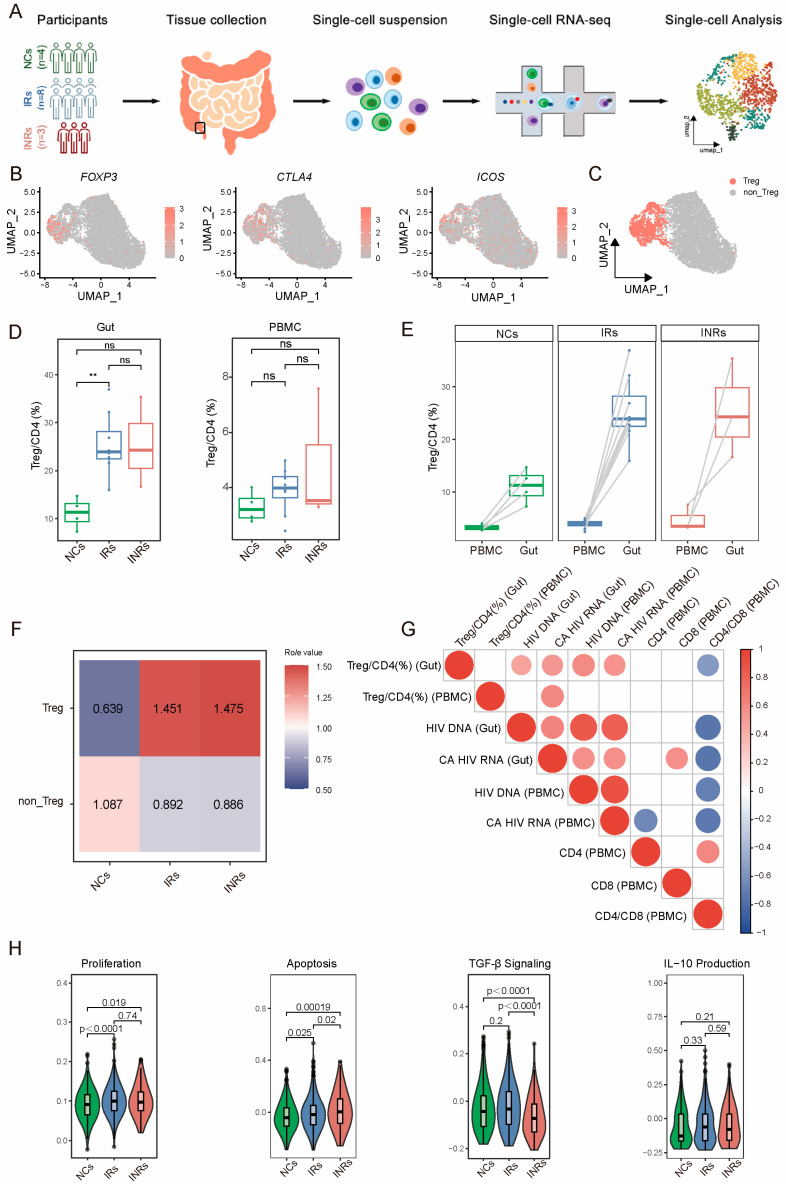
Single-cell gene expression profiling of Tregs derived from CD4^+^ T cells of the participants. (**A**) Schematic of scRNA-seq experimental procedure using ileocecal tissue from 4 NCs, 8 IRs and 3 INRs. (**B**) UMAP plots showing expression of Treg markers (*FOXP3*, *CTLA4, ICOS*). (**C**) UMAP plot identifying Tregs for more high-definition analysis. (**D**) Boxplot showing the proportions of Tregs in gut tissues (left panel) and peripheral blood (right panel) across the three groups. The Mann-Whitney U test was employed to assess statistical differences between groups. Significance levels are denoted as follows: ** *p* < 0.01; ns indicates non-significant results. (**E**) Comparison in Treg proportions between gut tissues and peripheral blood. (**F**) Enrichment scores of Tregs in the gut of NCs, IRs, and INRs were estimated based on the Ro/e score. (**G**) Correlation analysis of the Treg proportion in PBMCs or gut with the CD4^+^ T cell count, CD8^+^ T cell count, CD4/CD8 ratio, CA HIV RNA and HIV DNA. (**H**) Violin plot showing function-related scores of Tregs in the gut across the three groups. NCs: negative controls; IRs: immunological responders; INRs: immunological non-responders; Ro/e score, ratio of observed to expected cell numbers. UMAP: uniform manifold approximation and projection.

To investigate the immunological features of gut Tregs in HIV-infected individuals after ART, we performed scRNA-seq using ileocecal tissue samples from three groups: four NCs, eight IRs, and three INRs (Figure 1A). The characteristics of the enrolled individuals are detailed in Table 1. After quality control, we identified Tregs based on the expression of canonical markers (*FOXP3*, *CTLA4*, *ICOS*) and further categorized the CD4^+^ T cell population into Treg and non-Treg subsets (Figure 1B,C and Figure S1). Uniform manifold approximation and projection (UMAP) was used to visualize the subsets and their canonical gene markers (Figure S2A,B). A total of 1088 Tregs were obtained, comprising 388 cells for NCs, 545 cells for IRs and 155 cells for INRs were with a mean of 1046, 1123 and 1089 genes per cell, respectively.

We analyzed the proportion of Tregs in both gut tissues and peripheral blood across the three groups using paired PBMCs from the same individuals. The proportion of Tregs was higher in both gut tissues and peripheral blood of IRs or INRs, compared to NCs (Figure 1D). Additionally, the Treg proportion was consistently higher in gut tissues than in peripheral blood across all individuals (Figure 1E). In gut tissues, INRs exhibited a slightly elevated Treg proportion compared to IRs, while in peripheral blood, the Treg proportion was lower in the INRs than in IRs (Figure 1D). Ro/e analysis was performed; revealed significant enrichment of Tregs in HIV-infected individuals compared to the NCs (Figure 1F).

To explore the relationship between Treg proportion and disease progression, we correlated the Treg proportion with CA HIV RNA and HIV DNA levels in gut and peripheral blood, as well as peripheral blood CD4^+^ T cell count, CD8^+^ T cell count, and CD4/CD8 ratio. The gut Treg proportion was positively correlated with HIV reservoirs in both gut and peripheral blood (*p* < 0.05) and negatively correlated with the peripheral blood CD4/CD8 ratio (*p* < 0.05), suggesting that the increase in gut Treg proportion was correlated with disease progression (Figure 1G). Further analysis of Treg proliferation, apoptosis and functional capacity using gene set scoring (Supplementary Table S2) showed elevated proliferation scores in chronic HIV infection, with slightly higher scores in INRs than IRs. Apoptosis scores were also significantly higher in HIV-infected individuals, particularly in INRs compared to IRs, indicating a higher turnover rate in INRs. Additionally, compared to IRs, INRs exhibited significantly reduced scores in both the TGF-β signaling pathway and IL-10 production-related gene sets (Figure 1H). These findings suggest that despite a slight increase in Treg proportion, gut Tregs in INRs exhibit significant dysfunction.

### 3.2. Transcriptional Profiling of Gut Treg Subsets in NCs, IRs, and INRs

**Figure 2:**
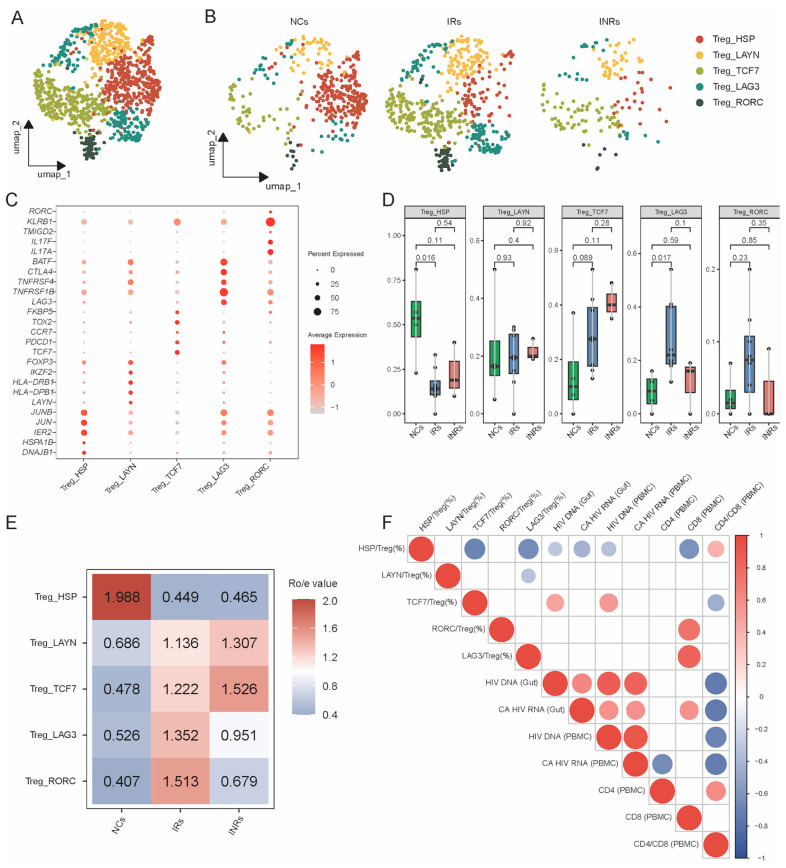
Heterogeneity of Treg cell subpopulations. (**A**) UMAP plots of Treg subpopulations. (**B**) UMAPs of Treg subpopulations across different groups. (**C**) Bubble plot showing the expression of classical marker genes across five Treg subpopulations. (**D**) Boxplot depicting the percentage of Treg subpopulations across distinct groups. (**E**) Enrichment scores of Treg subpopulations across the three groups. (**F**) Correlation analysis of the Treg subpopulation proportions and clinical indicators. UMAP: uniform manifold approximation and projection.

To better understand the heterogeneity of gut Tregs, we subdivided them into sub-clusters based on canonical gene markers. Five Treg subpopulations were identified: Treg_HSP (*JUNB*, *HSPA1B* and *DNAJB1*), Treg_LAYN (*IKZF2*, *HLA-DRB1* and *LAYN*), Treg_TCF7 (*TCF7*, *PDCD1* and *CCR7*), Treg_LAG3 (*BATF, CTLA4* and *LAG3*), and Treg_RORC (*RORC* and *IL17A*) (Figure 2A,C and Figure S2D). We assessed the frequency of these subpopulations across the three groups and found that Treg_HSP was significantly lower in IRs and INRs compared to NCs, while the proportion of other subsets was higher (Figure 2D,E). Specifically, the proportions of Treg_LAYN and Treg_TCF7 were increased in INRs compared to IRs, although not significantly. Correlation analysis with the counts of Treg_LAG3 and Treg_RORC were most abundant in the IRs. Correlation analysis with clinical indicators revealed that Treg_HSP frequencies were negatively correlated with viral reservoir indicators and CD8^+^ T cell counts but demonstrated positive correlation with the CD4/CD8 ratio (Figure 2F). Conversely, Treg_TCF7 frequency was positively correlated with viral reservoir indicators and negatively correlated with the CD4/CD8 ratio. The proportions of Treg_LAG3 and Treg_RORC were positively correlated with CD8^+^ T cell counts. Given the role of these subsets in regulating gut homeostasis [50], the decreased frequencies of Treg_LAG3 and Treg_RORC in INRs may reduce their ability to defend against microbiota and maintain tissue homeostasis.

**Figure 3:**
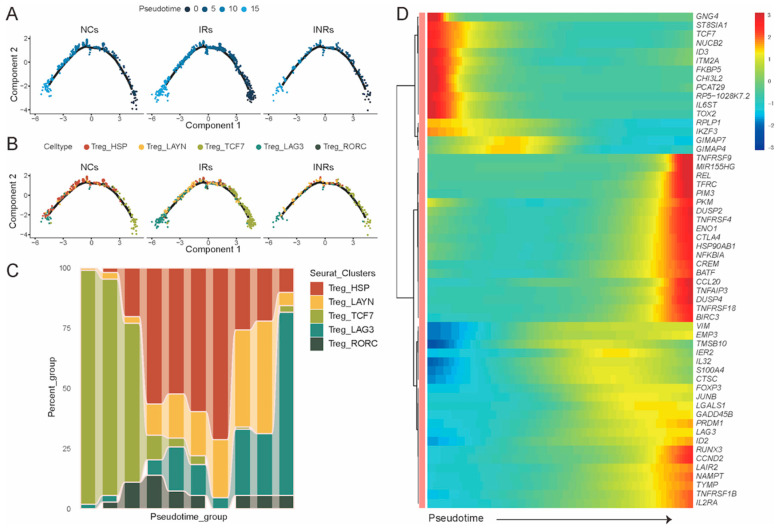
Lower differentiation of Treg subpopulations in INRs. (**A**,**B**) Pseudotime trajectory depicting the differentiation process of Treg subpopulations. (**C**) The patterns of cell density along with the pseudotime. (**D**) The patterns of gene expression along with the pseudotime.

To further investigate the differentiation status of gut Treg subsets, we constructed single-cell trajectories using the Monocle2 R package (version: 2.34.0). The subsets formed a continuous differentiation trajectory, starting with Treg_TCF7, transitioning through Treg_HSP and Treg_LAYN, and ending with Treg_RORC and Treg_LAG3 (Figure 3). A disruption in this differentiation trajectory was observed in INRs. At the onset of differentiation, Treg_TCF7 was the predominant subset, characterized by elevated expression of marker genes such as *TCF7*, *TOX2*, and *FKBP5*. Conversely, at the terminal stage, Treg_LAG3 emerged as the predominant subset, with increased expression of marker genes such as *LAG3*, *TNFRSF4, TNFRSF18*, *CTLA4*, and *BAFF*. These findings suggest that the altered differentiation trajectory and decreased frequencies of functional Treg subsets in INRs contribute to disease progression.

### 3.3. Reprogrammed Energy Metabolism of Gut Tregs in INRs

**Figure 4:**
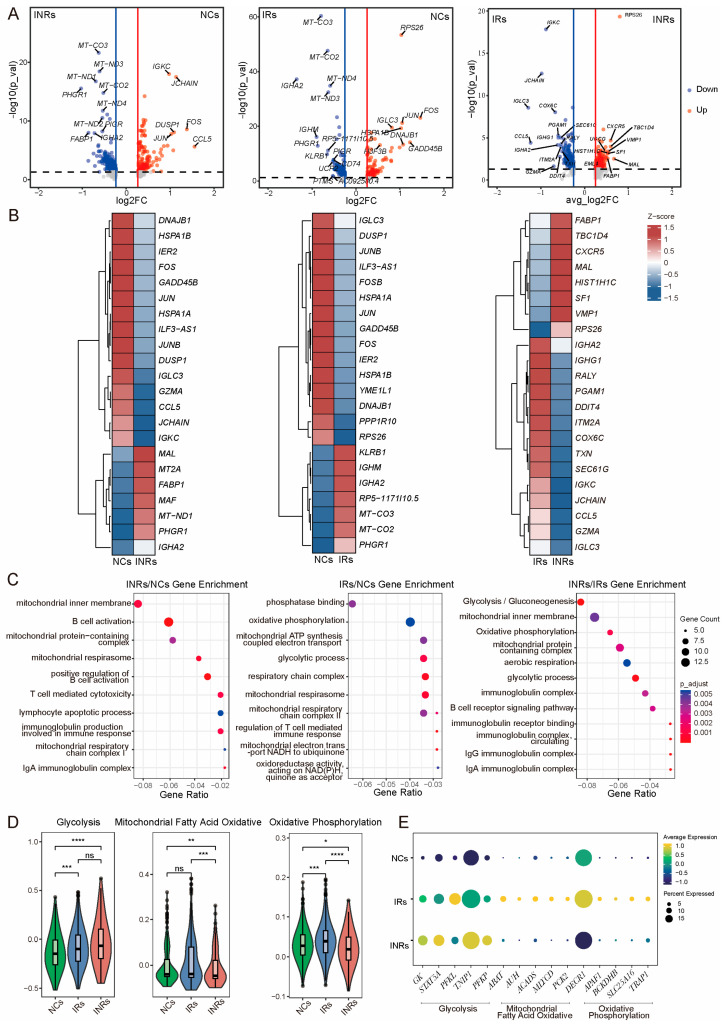
Reprogrammed energy metabolism of gut Tregs. (**A**) Volcano plots depicting the DEGs across comparisons of NCs/IRs, NCs/INRs, and INRs/IRs. (**B**) Cluster heatmap displaying the specific DEGs identified in the NCs/IRs, NCs/INRs, and INRs/IRs. Z-score standardization was applied to normalize the data by centering (subtracting the mean) and scaling (dividing by the standard deviation). (**C**) Bubble plot of GO enrichment analysis of the DEGs. “Count” indicates the number of DEGs enriched in pathway; “GeneRatio” indicates the ratio of enriched DEGs to background genes; “p.adj” indicates the *p*-value corrected by ‘BH’ method. (**D**) Violin plot showing metabolism-related scores for Tregs across the three groups. The Mann-Whitney U test was employed to assess statistical differences between groups. Significance levels are denoted as follows: * *p* < 0.05; ** *p* < 0.01; *** *p* < 0.001; **** *p* < 0.0001; ns indicates non-significant results. (**E**) Dot plot showing the expression levels of metabolism-related genes. DEGs: differentially expressed genes; GO: gene ontology; BH: Benjamini–Hochberg.

To explore the function of gut Tregs during immune reconstitution, we analyzed differences in gene expression profiles among the IRs, INRs, and NCs. A total of 333 significant DEGs were identified between NCs and IRs, 528 DEGs between NCs and INRs, and 369 DEGs between the IRs and INRs (Figure 4A,B). Gene enrichment analysis revealed that these DEGs were predominantly enriched in pathways related to glycolysis, OXPHOS, mitochondrial respiration, and the mitochondrial inner membrane (Figure 4C). Further analysis showed that glycolysis-related genes were upregulated in INRs compared to IRs, while mitochondrial fatty acid oxidation and OXPHOS pathways were downregulated, indicating a shift towards glycolysis over OXPHOS (Figure 4D). Key metabolic genes involved in these pathways exhibited similar trends (Figure 4E). These findings suggest that metabolic reprogramming of gut Tregs may play a vital role in shaping immune reconstitution outcomes in HIV-infected individuals undergoing ART.

### 3.4. Reduced in the Abundance of SCFA-Producing Bacteria and SCFA Concentration in INRs

**Figure 5:**
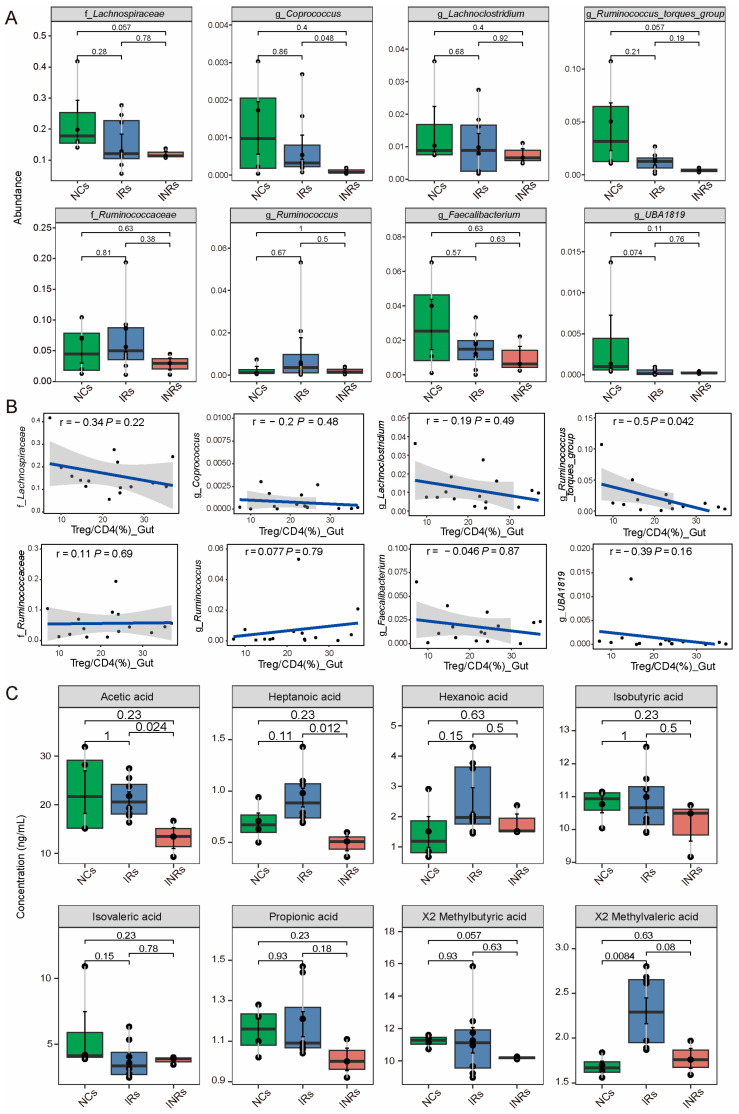
Decreased abundance of SCFA-producing bacteria and SCFAs concentrations in INRs. (**A**) The boxplots display the abundance of microbes across the groups. (**B**) The scatter plots show the relationship between the Treg proportion in gut tissues with the abundance of gut microbiota. “R” denotes the Spearman correlation coefficient, and “P” represents the *p*-value from the statistical test. (**C**) Boxplots show the concentrations of SCFAs among the groups. SCFAs: short-chain fatty acids. UNS: un-stimulated.

Previous studies have demonstrated that SCFAs, produced by gut microbiota, play a crucial role in regulating Treg homeostasis in the gut [36]. We analyzed the abundance of SCFA-producing gut microbiota and found that families such as *Lachnospiraceae* and *Ruminococcaceae*, and genera such as *Coprococcus*, *Lachnoclostridium*, *Ruminococcus torques*, *Ruminococcus*, *Faecalibacterium*, and *UBA1819* were more abundant in NCs than in IRs and INRs (Figure 5A). Notably, the abundance of these microbiota was consistently lower in INRs compared to IRs. Correlation analysis revealed a significant negative correlation between the Treg proportion and the abundance of *Ruminococcus torques* (*r* = −0.55, *p* < 0.05), while other microbiota showed a negative trend without statistical significance (*p* > 0.05). Indeed, SCFA production is better represented in serum than in feces [51]. We therefore additionally quantified SCFA concentrations in the circulation and found that most SCFAs, except isovaleric acid, were decreased in INRs compared to IRs, with significant differences observed for acetic acid and heptanoic acid (Figure 5C).

### 3.5. SCFA Stimulation Restores Treg Dysfunction

**Figure 6:**
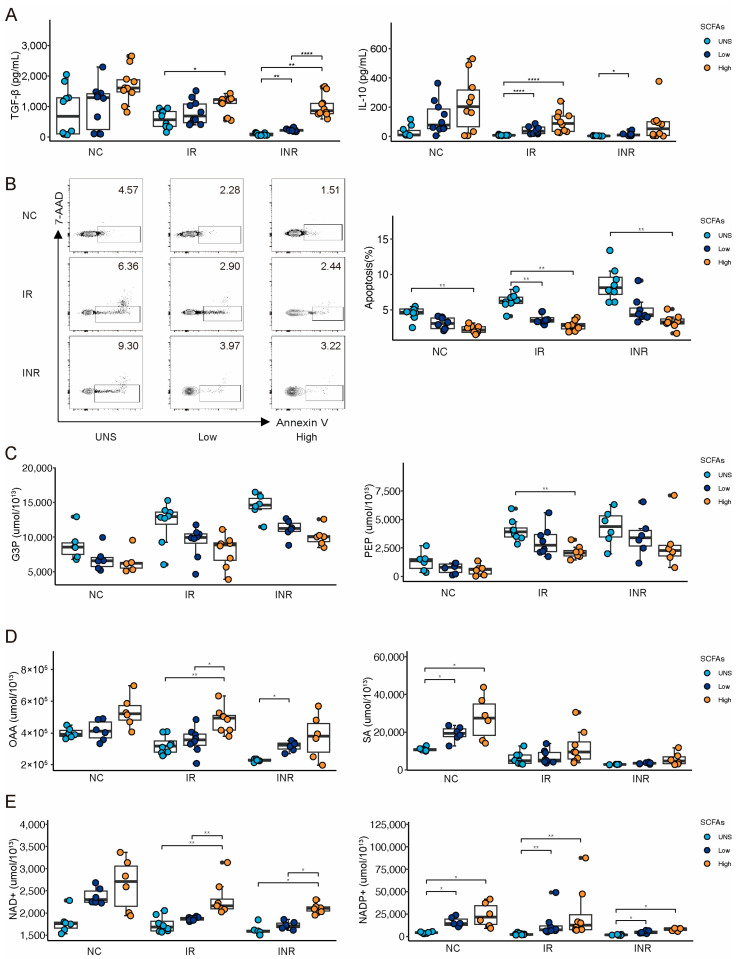
SCFA suspension restores Treg function in INRs. (**A**) Boxplots show relative abundance of TGF-β (left) and IL-10 (right) secreted by Tregs following the administration of low or high concentrations of SCFAs. (**B**) Representative flow cytometry contour plots (left) and the box and scatter plots (right) illustrating the percentage of Treg early apoptosis. (**C**) Boxplots show changes in glycolytic metabolites G3P (left) and PEP (right) with low or high SCFA concentration stimulation. (**D**) Boxplots show changes in TCA cycle metabolites OAA (left) and SA (right) under different stimulations. (**E**) Boxplots show changes of the energy metabolism-related metabolites NAD^+^ (left) and NADP^+^ (right) under different situations. Different colors correspond to varying SCFA concentrations. G3P: glyceraldehyde-3-phosphate; PEP: phosphoenolpyruvate; OAA: oxaloacetate; SA: succinic acid; CA: tricarboxylic acid; NAD^+^: nicotinamide adenine dinucleotide; NADP^+^: nicotinamide adenine dinucleotide phosphate. Significant differences are indicated by * *p* < 0.05; ** *p* < 0.01; **** *p* < 0.0001.

To validate the effects of SCFAs on Treg function, we isolated CD4^+^CD25^+^CD127^−^ Tregs from peripheral blood samples collected from 44 individuals (14 NCs, 16 IRs, and 14 INRs) and cultured them with low or high doses of SCFA mixtures for 3 days. We found that TGF-β and IL-10 production was significantly lower in HIV-infected patients than in NCs, particularly in INRs (Figure 6A). SCFA stimulation consistently promoted the production of TGF-β and IL-10 across all groups, indicating enhanced Treg cytokine production activity in the presence of SCFAs.

Next, we evaluated the impact of SCFAs on Treg apoptosis using flow cytometry. SCFA stimulation consistently reduced Treg early apoptosis across all groups, although early apoptosis levels remained highest in INRs, intermediate in IRs, and lowest in NCs (Figure 6B). This highlights the functional impairments in Tregs among INRs.

Given the observed metabolic reprogramming in Tregs [52,53], we assessed the levels of glycolysis and OXPHOS-related metabolites in SCFA-stimulated Tregs using mass spectrometry. Glycolytic intermediates such as glyceraldehyde-3-phosphate (G3P) and phosphoenolpyruvate (PEP) decreased with increasing SCFA concentrations across all groups, remaining highest in INRs, intermediate in IRs, and lowest in NCs (Figure 6C). Conversely, tricarboxylic acid (TCA) cycle intermediates such as oxaloacetate (OAA) and succinic acid (SA) increased with higher SCFA concentrations, although their levels were consistently lower in INRs compared to IRs and NCs (Figure 6D). Finally, we examined changes in OXPHOS-related metabolites and found that nicotinamide adenine dinucleotide (NAD^+^) and nicotinamide adenine dinucleotide phosphate (NADP^+^) increased with higher SCFA concentrations, with the greatest increases observed in NCs, followed by IRs, and the smallest increases in INRs (Figure 6E). These results suggest that exogenous SCFA supplementation can restore the preferential utilization of glycolysis over OXPHOS in INRs, thereby restoring Treg function and metabolism.

## 4. Discussion

The gut is a complex system that interacts with the external environment, harboring a diverse community of microorganisms and continually exposed to a wide range of food antigens. Tregs play a vital role in the gut by regulating immune responses and preventing excessive reactions to these microorganisms and antigens, thereby maintaining immune tolerance [54]. While Tregs in peripheral blood are important, those in the gut are particularly significant due to the gut’s unique immune system and microenvironment. They play a crucial role in preserving gut homeostasis and safeguarding overall gut health [54,55,56]. Additionally, the gut is a primary site for HIV infection and replication. In HIV-infected individuals, significant alterations in the gut microbiota have been observed, particularly in INRs compared to IRs [35,57]. Previous studies have shown that prebiotic and probiotic therapies can boost immune reconstitution and decreases inflammation in both HIV-positive patients undergoing treatment and infected macaques [58,59,60,61,62]. Furthermore, the failure to restore CD4^+^ T cell counts in INRs has been attributed to impairments in the survival and functionality of Tregs, leading to uncontrolled proliferation, immune exhaustion, and increased rates of cell death [63]. While extensive research has focused on Tregs in the peripheral blood of HIV-infected individuals, much remains unknown regarding the characteristics of gut Tregs, their relationship to immune reconstitution and whether bacterial dysbiosis and metabolites contribute to these outcomes. In this study, we used scRNA-seq technology to conduct an in-depth analysis of the cellular characteristics of gut Tregs in patients who either successfully or unsuccessfully underwent immune reconstitution following long-term ART. Our results revealed that INRs showed a slightly elevated proportion of Tregs within CD4^+^ T cells, in contrast to IRs, but demonstrated impaired Tregs functionality in the gut. Specifically, Tregs in INRs exhibited a heightened reliance on glycolysis for energy production, compared to those in IRs, accompanied by a significant decline in their OXPHOS capacity. The results of the present study indicate that these alterations in gut Tregs were intricately associated with the obvious decrease in SCFAs-producing bacteria and SCFAs concentration in INRs, suggesting a crucial role of SCFAs in immune reconstitution after HIV infection.

In our study, we observed that the proportion of Tregs within CD4^+^ T cells was consistently higher in the gut compared to peripheral blood, regardless of group membership. Notably, both IRs and INRs had an elevated proportion of Tregs in the gut compared to NCs, supporting previous findings of abnormal Treg upregulation in the gut during HIV infection [64,65]. This aligns with findings from other studies indicating that, despite ART typically restoring immune reconstitution in the peripheral blood of HIV-infected individuals [66], the frequency of Tregs remains stable in gut tissues, even when ART is initiated early [67]. This may be attributed to an abnormal upregulation of Tregs that migrate to the gut during HIV infection [68], or despite a high level of early apoptosis among Tregs in INRs, their proportion remains slightly elevated. Additionally, some reports indicate that the percentage of Tregs increases steadily as the disease progresses [69]. Our previous immunohistochemical results have shown that the proportion of Tregs within CD4^+^ T cells is higher in INRs compared to IRs. Nevertheless, contrasting reports indicate a lower frequency of Tregs in the gut mucosa [70] and lymphatic tissues [40] of INRs. This apparent discrepancy may be due to multiple factors, including the relatively small sample size in this study, potential inconsistencies in patient selection criteria across studies, and variations in the Treg cell markers used. Future studies will endeavor to resolve these discrepancies and enhance the reliability of the findings by conducting larger study cohorts, employing standardized patient selection criteria, and utilizing uniform and consistent parameters for defining Tregs.

We observed a notably elevated proportion of Treg_HSP in NCs compared to HIV-infected individuals. This discrepancy likely reflects the inclusion of NCs with concurrent inflammatory comorbidities, which may induce upregulation of proteins that respond to damaging stress and maintain proteostasis in these individuals. Furthermore, we delineated two subsets, namely Treg_LAG3 and Treg_RORC, corresponding to the main subpopulations of gut Tregs, which regulate inflammation and mediate tolerance to microorganisms, respectively [50]. The proportions of these two subsets were observed to be lower in individuals with INR compared to those with IR. Also, our data revealed a correlation between the percentage of these subsets and disease progression, suggesting that the altered distribution of Treg subsets may impact the homeostasis of the gut microenvironment. High levels of *TCF7*, *TOX2*, and *FKBP5* in Treg_TCF7 and high levels of *FOXP3*, *IL2RA*, *CTLA4* and *TNFRSF18* in Treg_LAG3 were observed. We identified Treg_TCF7 as the starting point of differentiation and Treg_LAG3 as the end by cellular trajectory analysis. In addition, we found a differentiation gap within the Treg cell subpopulation in INRs. The present study displayed an increased proportion of Treg_TCF7 but a decrease percentage of Treg_LAG3 and disrupted differentiation of Treg cell subsets, indicating that the alteration of Treg cell subsets may affect immune reconstitution after HIV infection.

Tregs play a pivotal role in immune tolerance by secreting inhibitory cytokines, such as IL-10 and TGF-β, to maintain immune homeostasis [71,72]. In this study, we observed that Tregs in the gut of INRs exhibited decreased secretion of IL-10 and TGF-β, despite an increased turnover rate, compared with IRs. This increased renewal of Tregs, which is associated with the disease progression, may be induced by higher inflammation-driven microbiota dysbiosis. Furthermore, cellular energy metabolism plays a crucial role in determining the fate and function of immune cells, including Tregs [52,73]. In our study, we compared the major energy metabolism of Tregs between IRs and INRs. Our findings indicated that, in contrast to Tregs from IR individuals, gut Tregs from INR individuals display a markedly increased level of glycolysis and a significantly reduced level of OXPHOS. Prior research has shown that glycolysis, while essential for sustaining Treg cell proliferation, inhibits their functional capacity. Conversely, an elevation in OXPHOS pathway within Tregs is associated with enhanced functional capabilities [52,63,74]. Previous studies have confirmed that OXPHOS may serve as a potential target for HIV antiretroviral therapy [75]. As such, the enhanced turnover rate but reduced functionality of gut Tregs in INRs may be associated with their increased reliance on glycolysis and decreased utilization of OXPHOS during energy metabolism.

SCFAs, which are fatty acids containing fewer than six carbon atoms, are primarily produced by gut microbiota through the fermentation of dietary fiber [76]. These acids play a crucial role in maintaining Tregs homeostasis, thereby regulating the function of gut Tregs by influencing metabolism and energy stabilization [36,39]. In addition, significant reductions in SCFAs are associated with inflammation and might be used as indicators to predict morbidity and mortality in people with HIV [51]. In this study, we observed decreased levels of SCFAs in the peripheral blood and a reduction in the abundance of SCFA-producing microbiota in INRs. Consequently, we hypothesize that the increased number but decreased functionality of Tregs in INRs may be a consequence of altered SCFA and microbiota composition. Our in vitro experiments confirmed that SCFAs enhance the OXPHOS pathway while suppressing the glycolysis pathway in Tregs from both IRs and INRs. As a result, the observed decrease in SCFAs may contribute to elevated glycolysis and reduced OXPHOS, ultimately increasing the Treg turnover rate but decreasing their functional capacity. More specialized experiments are needed to explore the altered characteristics of gut Tregs in INRs in greater depth. Recently, Wahl et al. developed a germ-free humanized mouse model and found that resident microbiota promote the establishment of mucosal HIV infection [77]. It is well worth determining the relationship and the detailed mechanisms among gut microbiota, gut Tregs and HIV immune reconstitution using the above-mentioned mouse model.

This study has several limitations. First, the difficulty in obtaining gut tissue biopsies resulted in a relatively small sample size. Future studies should validate these results in larger cohorts. Second, the mechanistic investigation remains preliminary; future work should employ germ-free humanized mouse models to elucidate the interactions between gut microbiota, gut Treg cells, and systemic immune reconstitution in HIV. Third, the clinical relevance of SCFA-mediated Treg modulation for immune reconstitution remains unverified. Future therapeutic strategies—including dietary fiber supplementation or engineered probiotics to boost SCFA levels, combined with metabolic pathway modulation in Tregs—may offer promising approaches to address inadequate immune recovery in people living with HIV.

In summary, our study indicates that Tregs in the gut of INRs exhibit an elevated turnover rate but impaired suppressive function. This may be correlated with enhanced glycolysis and decreased OXPHOS within these Tregs. Administering SCFAs to modulate the metabolic pathways of Tregs in INRs emerges as a potentially promising strategy to address immune reconstitution failure following HIV infection.

## Figures and Tables

**Table 1:** Characteristics of enrolled participants for scRNA-seq.

	NCs (n = 4)	IRs (n = 8)	INRs (n = 3)	*p*_Value
Age (years)	45 (39–46)	40 (30–48)	45 (40–49)	0.497
Gender (Male/Female)	4/0	8/0	3/0	
CD4^+^ T cell count (cells/µL)	919 (844–980)	861 (730–1006)	135 (133–138)	0.0206 *
CD8^+^ T cell count (cells/µL)	471 (424–527)	1033 (877–1370)	482 (479–597)	0.0015 **
CD4/CD8 ratio	2.01 (1.83–2.13)	0.79 (0.73–0.92)	0.27 (0.24–0.28)	<0.0001 ****
Nadir CD4^+^ T cell count (cells/µL)	-	352 (299–382)	24 (10–53)	0.0121 *
ART duration (years)	-	4 (3–6)	4(3–5)	0.63
ART regimen (%)				1
2NRTIs+1NNRTIs	-	5 (62.5%)	2 (66.7%)	
2NRTIs+1INSTIs	-	3 (37.5%)	1 (33.3%)	

The data are presented as the median with the interquartile range. All patients exhibited viral loads below the detectable level. Kruskal–Wallis test and Mann–Whitney U test were used to compare continuous variables. Fisher’s exact test was used to compare categorical variables. Significant differences are indicated by * *p* < 0.05; ** *p* < 0.01; **** *p* < 0.0001. NCs, HIV-negative controls; IRs, immunological responders; INRs, immunological non-responders; NRTIs, nucleoside reverse transcriptase inhibitors; NNRTIs, non- nucleoside reverse transcriptase inhibitors; INSTIs, integrase strand transfer inhibitors. -, not available.

## Data Availability

The raw data for single-cell RNA sequencing reported in this publication can be accessed under the Chinese Academy of Sciences (GSA-Human: HRA007250) and are publicly accessible at https://ngdc.cncb.ac.cn/gsa-human (accessed on 8 October 2024).

## Supplementary Materials

The following supporting information can be downloaded at https://www.mdpi.com/article/10.3390/cells14151164/s1, Figure S1: Quality control (QC) of the scRNA-seq data; Figure S2: Single-cell Characterization of Treg Heterogeneity; Figure S3: Flow-cytometry gating strategy for Tregs; Table S1: Characteristics of participants for in vitro experiment; Table S2: List of gene sets for cell function scoring.
