# Supplementary material for: Dysfunction and Metabolic Reprogramming of Gut Regulatory T Cells in HIV-Infected Immunological Non-Responders
Source: Cells. 2025 Jul 29;14(15):1164. doi: 10.3390/cells14151164 (PMC12346641; doi:10.3390/cells14151164)
Supplement: Supplementary file 1 [file cells-14-01164-s001.zip › cells-3750274-supplementary.pdf]

Supplementary Information

Table S1. Characteristics of participants for in vitro experiment.

	NCs (n = 14)	IRs (n = 16)	INRs (n = 14)	P_value
Age (years)	41（39-42）	43 (40-46)	48 (37-50)	0.104
Gender (Male/Female)	14/0	16/1	14/3	
$CD4^+$ T cell count(cells/uL)	690.5 (629-849.75)	677 (609.5-913.5)	281 (263-308)	<0.0001****
$CD8^+$ T cell count (cells/uL)	468 (447-97)	851 (520-1162)	688 (527-892)	0.001**
CD4/CD8 ratio	1.48 (1.41-1.70)	0.95 (0.65-1.49)	0.43 (0.34-0.54)	<0.0001****
Nadir $CD4^+$ T cell count (cells/μL)	-	337 (320-357)	27 (18-40)	<0.0001****
ART duration (years)	-	7 (4-8)	6 (5, 7)	0.9
ART regimen (%)				0.157
2NRTIs+1NNRTIs	-	15 (93.8%)	10 (71.4%)	
2NRTIs+1INSTIs	-	1 (6.3%)	4 (28.6%)	

The data are shown as the median with the interquartile range. All patients exhibited viral loads below the detectable level. Kruskal-Wallis test and Mann–Whitney U test were used to compare continuous variables. Fisher's exact test was used to compare categorical variables. Significant differences are indicated by **P < 0.001; ****P < 0.00001. NCs, HIV-negative controls; IRs, immunological responders; INRs, immunological non-responders; NRTIs, nucleoside reverse transcriptase inhibitors; NNRTIs, non- nucleoside reverse transcriptase inhibitors; INSTIs, integrase strand transfer inhibitors. -, not available.

Table S2. List of gene sets for cell function scoring.

Terms	Gene Sets
IL-10 Production	*PRG2, SASH3, NFKBIZ, PRKCD, G6PDX, FOXP3, IDO1, TLR4, EPX, IL21, CD83, FCGR2*
TGF-β Signaling Pathway	*TGFB1, TGFB1I1, SMAD1, SMAD9, HDAC1, HDAC2, SK2L, LEFTY1, RBBP7, SDCBP, SPI1, SRC, SPRY1, GIPC1, FAM89B, APPL1, SNX25, PTK2, PTPRK, PML*
Proliferation	*ABL1, AGER, AIF1, ANXA1, ARG1, ARG2, BAX, BCL6, BID, BMI1, BMP4, CASP3, CBLB, CCND3, CD1D, CD3E, CD6, CD28, CD80, CD86, TNFSF8, CD40LG, CD70, CD81, CD151, CEBPB, CLC, CR1, CTLA4, CTNNB1, CTPS1, CD55, DHPS, DLG1, DOCK2, EFNB1, ELF4, EPO, ERBB2, FKBP1B, FOXJ1, FYN, LRRC32, NCKAP1L, HLA-A, HLA-DMB, HLA-DPA1, HLA-DPB1, HLA-DRB1, HLA-E, HLA-G, HMGB1, HES1, IGF1, IGF2, IGFBP2, IHH, IL1A, IL1B, IL2, IL2RA, IL4, IL6, IL6ST, IL10, IL12B, IL12RB1, IL15, TNFRSF9, IL18, IDO1, IRF1, JAK2, LEP, LGALS3, LGALS9, LIPA, LMO1, CD46, KITLG, MSN, NCK1, PNP, P2RX7, PAWR, PIK3CG, PLA2G2A, PLA2G5, PPP3CA, PPP3CB, PRKAR1A, PRKCQ, PRNP, PSMB10, PTPN6, PTPRC, RAC2, RPS3, RPS6, CCL5, CCL19, XCL1, SDC4, SFTPD, SHH, SLAMF1, SLC4A2, SLC7A1, SLC11A1, SOS1, SOS2, SPN, SPTA1, STAT5B, SYK, PRDX2, TFRC, TGFBR2, TNFRSF1B, TP53, TRAF6, TNFSF4, TNFRSF4, TYK2, VCAM1, ZAP70, ZP3, MAD1L1, NCK2, PDE5A, TNFSF14, TNFSF9, TNFRSF14, RIPK2, FADD, TNFSF18, SH2D2A, DNAJA3, DLG5, MAPK8IP1, TSPAN32, RASGRP1, EBI3, LILRB2, BTN2A2, GPNMB, TNFSF13B, LILRB1, MALT1, LILRB4, RIPK3, BTN3A1, GLMN, HHLA2, CORO1A, VSIG4, ICOSLG, NCSTN, SCRIB, PTPN22, PLA2G2D, TNFRSF21, PYCARD, CD274, CD209, FOXP3,*

Terms	Gene Sets
	ZBTB7B, IL23A, IL20RB, WNT4, SASH3, RC3H2, LMBR1L, CRTAM, TWSG1, PELI1, SH3RF1, GPAM, IL21, PLA2G2F, RASAL3, ARMC5, PDCD1LG2, CD276, NDFIP1, DOCK8, ITCH, CARD11, HAVCR2, TNFRSF13C, TMIGD2, RC3H1, IL23R, IL27, IL4I1, CCDC88B, LGALS9B, CLEC4G, LGALS9C, CCR2, CD24
Apoptosis	*BCL2A1, BCL2L1, IL6R, IL7, NFE2L2, PDK1, PDK2, RAF1*
Glycolysis	*GK, STAT5A, ZNF654, RORA, SMAD3, SMAD4, TNIP1, WDR1, RNF24, EP300, CD44, HSP90B1*
Oxidative Phosphorylation	*ACADSB, ACAT1, AIFM1, ATP5B, ATP5G1, ATP5H, ATP5J, COX11, COX5B, COX6C, COX7A1, COX7C, CRAT, CYC1, DAP3, DBT, DECR1, DIABLO, DUT, ECHS1, ECI1, FDXR, FIBP, GCDH, GOT2, MIPEP, MRPL11, MRPL12, MRPL18, MRPL42, MRPS15, MRPS16, MRPS18C, MRPS28, MRPS33, NDUFA10, NDUFA5, NDUFAB1, NDUFB1, NDUFB2, NDUFB3, NDUFB5, NDUFB6, NDUFB8, NDUFS6, NDUFS8, NME4, PCCB, PCK2, PDHA1, PDHB, SDHB, SLC25A1, SLC25A17, SUCLG1, SURF1, TFAM, TIMM13, TSPO, TUFM, UQCR11, VDAC3, WARS2, ABAT, ABCB6, ABCB7, ABCB8, ACADL, ACADM, ACADS, ACADVL, ACP6, ALAS1, ALDH2, ALDH4A1, AMACR, AMT, APAF1, ATP5D, ATP5S, ATPIF1, BAX, BCAT2, BCKDHB, BDH1, CA5A, CA5B, CASP8, CASQ1, CKMT2, CLPP, CNOT7, COQ7, COX10, COX15, COX6A2, CYP11A1, CYP27B1, DGUOK, DHODH, DNASE2, ENDOG, ETFA, ETFB, FARSA, FDX1, FXN, GCAT, GCK, GLS2, GSR, HADH, HK1, HPS1, HSPD1, HSPE1, HTATSF1, IDH3A, IVD, LARS2, LMF2, LYPLA2, MAOB, MAST1, MBD3, MCAT, ME2, ME3, MLYCD, MRPL19, MRPL2, MRPL3, MRPL33, MRPL34, MRPL4, MRPL48, MRPS11, MRPS12, MRPS14, MRPS34, MRPS35, MTCH2, MTHFD1, MTIF2, MTRF1, MTX2, NCAPH2, NDUFA2, NDUFB7, NGFRAP1, OTC, OXCT1, PCCA, PIN4, POLG2, POLRMT, PPOX, PRDX3, SARDH, SCP2, SDHC, SLC25A10, SLC25A11, SLC25A12, SLC25A15, SLC25A16, SLC25A22, SLC25A40, SPG7, SSBP1, STARD3, TBCB, TFB1M, TIMM44, TK2, TONSL, TST, TXN2, TXNRD2, UCP1, UCP2, UCP3, UQCRC1, ZNF33B, ACAA1, ACSL6, ADCK2, AGMAT, AKAP1, ALDH18A1, ALDH6A1, ATP6V1B1, ATP7B, ATP8A1, ATPAF2, AUH, BAD, BIK, BOP1, C1QBP, C21orf2, CAD, CAT, CHDH, COX18, CROT, CRYZ, CRYZL1, CYP2E1, ECI2, EHHADH, ERBB2, FAM3A, G6PD, GAL3ST1, GALR3, GIMAP5, GOT1, GPT, GSTO1, HCLS1, HINT2, HSD17B10, HSD3B2, LCAT, LRPPRC, MB, MCCC1, MCCC2, MCEE, MLH3, MRPL40, MRPL41, MRPS17, MTOR, MTX1, MYCBP, NDUFAF1, NDUFB10, NDUFB9, NDUFS5, NDUFS7, NDUFV1, NIPSNAP1, OGDH, OGG1, PKLR, PLA2G1B, POLR1B, POLR3B, PPID, PRDX5, PRKCD, PYGB, REXO2, RPAIN, SCO2, SDS, SFXN2, SHMT1, SLC16A5, SLC25A39, SLC40A1, SLC9A5, SNCA, TFB2M, TP53AIP1, TRAP1, UNG, UQCRQ, UROS, YARS2*
Mitochondrial Fatty Acid Oxidation	*ACAT1, ALDH7A1, ALDH6A1, ACADSB, ABAT, AUH, ACADS, MCCC2, MLYCD, ACOX3, ACYP2, PCK2*

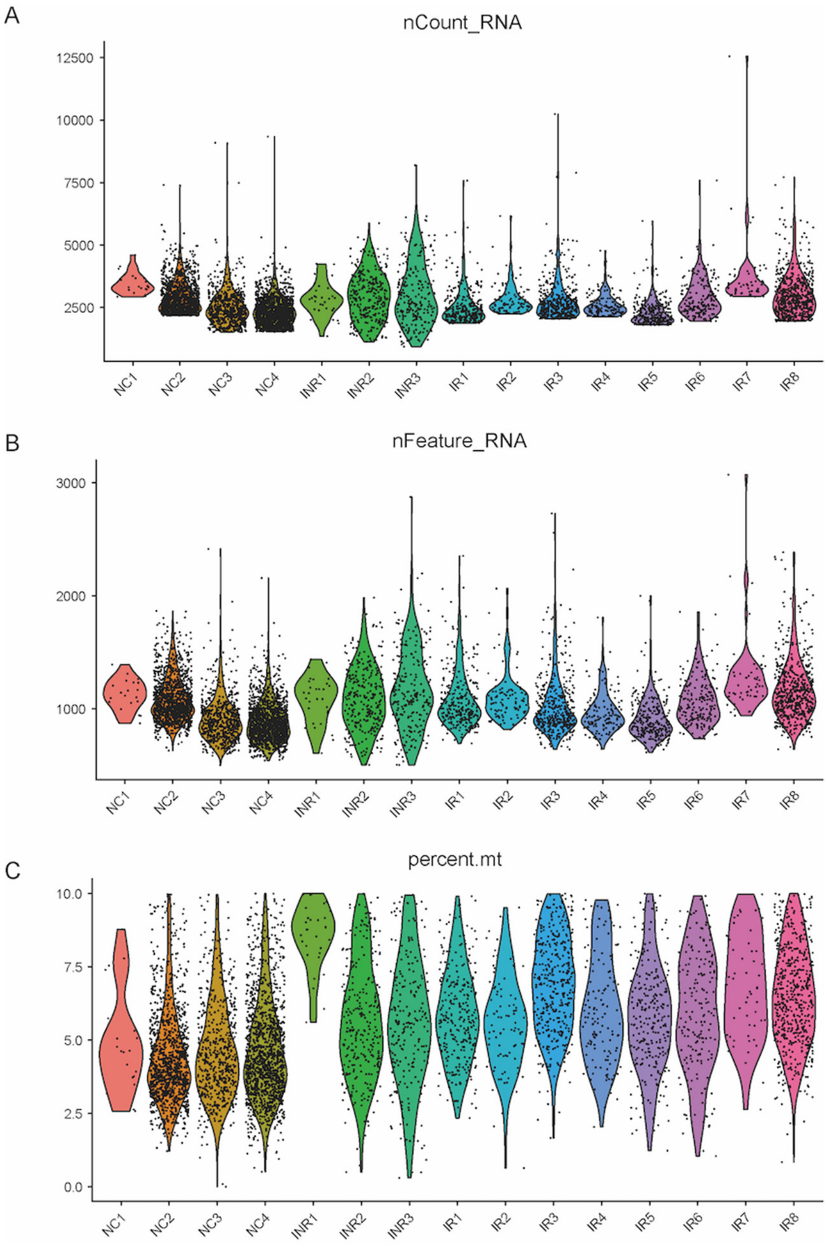

Figure S1. Quality control (QC) of the scRNA-seq data. A. Violin plot show the nCount_RNA of each sample, nCount: unique molecular identifiers (UMIs); B. Violin plot show the nFeature_RNA of each sample, nFeature: number of genes; C. Violin plot show the percent.mt of each sample, percent.mt: percentage of mitochondrial genes.

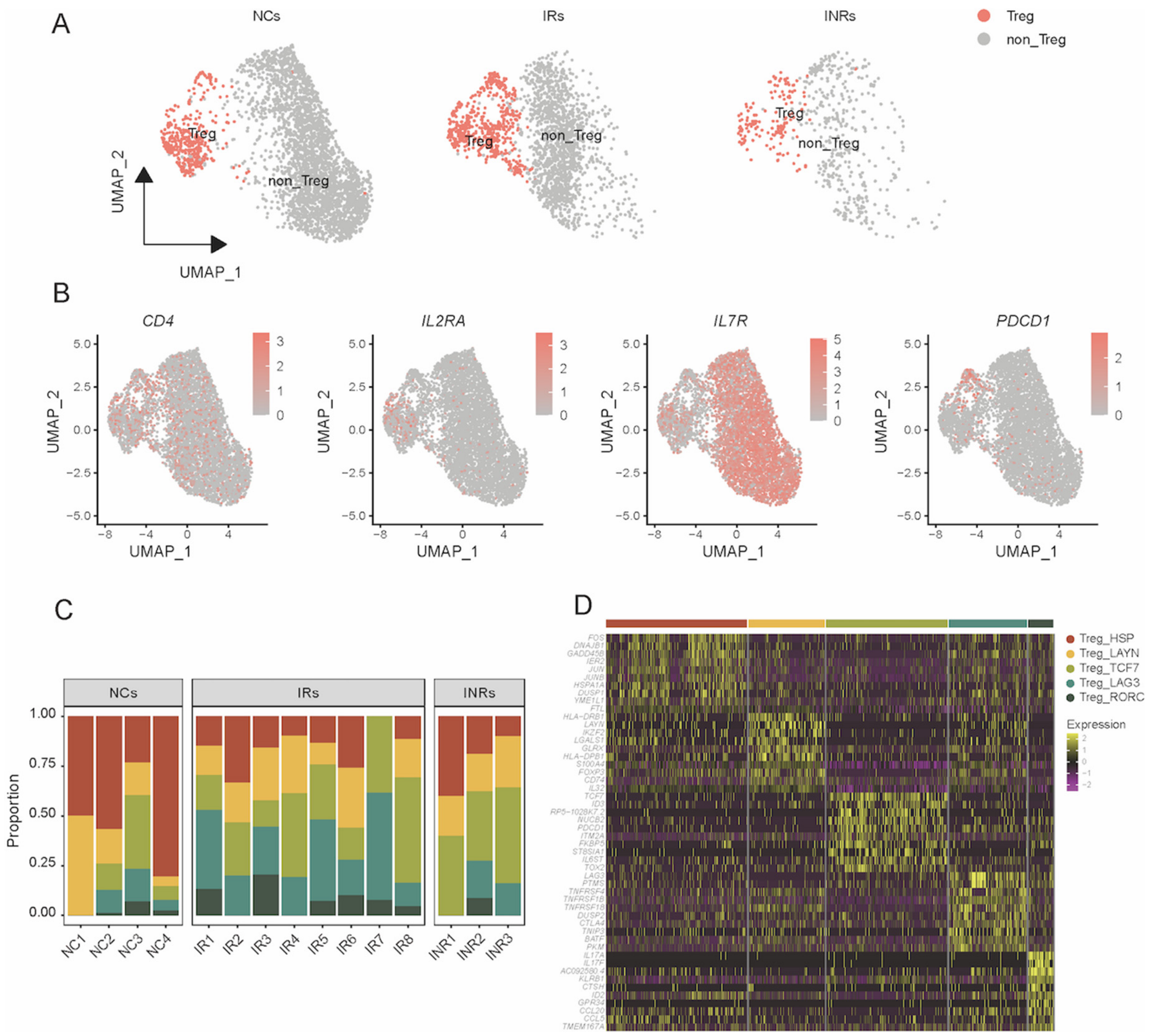

Figure S2. Single-cell Characterization of Treg Heterogeneity. A. UMAP visualization of Treg populations across different groups. B. Feature plots displaying the expression distribution of key Treg markers (*CD4*, *IL2RA*, *IL7R*, and *PDCD1*) across the UMAP projection. C. Quantitative analysis of Treg subset proportions within the total Treg population, presented as percentage distribution across individual samples. D. Heatmap visualization of differentially expressed genes characterizing distinct Treg subsets. The top-ranked subset-specific genes are shown with hierarchical clustering.

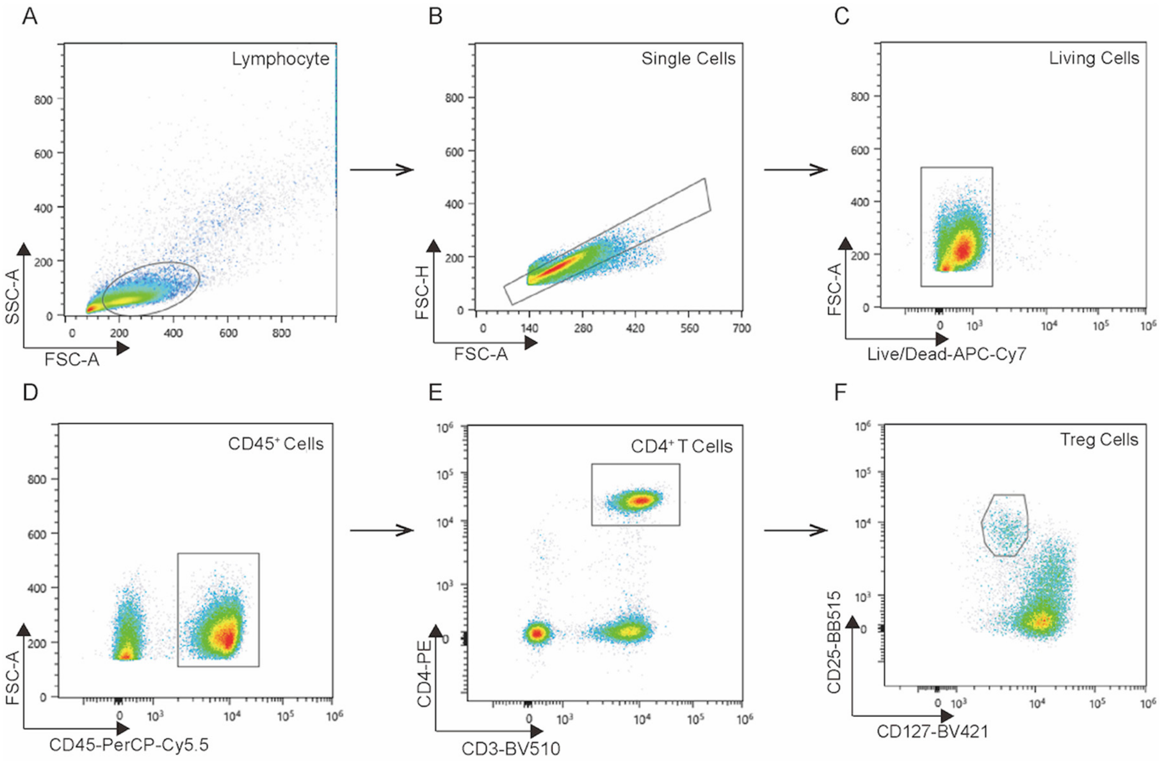

Figure S3. Flow-cytometry gating strategy for Tregs. Gating hierarchy of a representative sample. A. In SSC-A versus FSC-A, identification of the cloud of cell-like events; B. In FSC-H versus FSC-A, identification of singlets (single cell signals); C. In FSC-A versus Live/Dead-APC-Cy7, identification of living cells (living cell signals); D. In FSC-A versus CD45-PerCP-Cy5.5, identification of $CD45^+$ cells; E. In CD4-PE versus CD3-BV510, identification of $CD4^+$ cells; F. In CD25-BB515 versus CD127-BV421, identification of Treg cells.
